# Quest of Soil Protists in a New Era

**DOI:** 10.1264/jsme2.ME3202rh

**Published:** 2017-06

**Authors:** Jun Murase

**Affiliations:** 1 Graduate School of Bioagricultural Sciences, Nagoya UniversityFuro-cho, Chikusa, Nagoya 464–8601Japan

The taxon Protista was originally given by Ernst Haeckel in 1866 and included all unicellular organisms either prokaryotic or eukaryotic. In the modern view, the protists are eukaryotic organisms of unicellular organization, and thus the term embraces classical protozoa, unicellular phototrophic organisms such as diatoms, and lower unicellular fungi (28). Due to the extreme diversity, the taxonomic system of protists has been revised several times according to new knowledge and concepts (11, 14, 65). Protists are currently recognized to be paraphyletic or even polyphyletic and to be widely distributed in all of the five major supergroups—like kingdoms—under the eukaryote classification proposed by the International Society of Protistologists (1).

Protozoa are ‘animal-like’ protists that prey on other organisms. However, the term protozoa is no more (or at least much less) used due to the fact that some protozoa such as the euglenoids do photosynthesis as well. Different terms like ‘phagotrophic protists’, ‘heterotrophic protists’, and even just ‘protists’ are used to refer to protozoa depending on the context: the term ‘phagotrophic protists’ is used in this article. Among the members of protists in soil, phagotrophic protists are one of the groups that have been long and best studied. The composition and distribution of the phagotrophic protists (protozoan fauna) with different morphotypes—ciliates, flagellates, and amoeba—in soil was studied through a culture-based technique in 1920’s (55). Phagotrophic protists play a role in soil as bacterivores and their ecological significance had been demonstrated contemporaneously with the concept ‘microbial loop’ in the marine environments (6, 12, 13). Despite the long history of soil protozoology or protistology and the fact that the diversity of protists in soil would be as high as that in aquatic environments (7, 24), soil protists including phagotrophs are much less studied than their aquatic counterparts and this gap is increasing (26).

## Advent of molecular approaches in soil protistology

The advent of molecular approaches has been revolutionizing microbial ecology (33, 50). The same is true for the ecology of soil protists a little behind the prokaryotic ecology (51). The comprehensive surveys by high-throughput sequencing (HTS) approaches have demonstrated the great diversity of protists in soil and illuminated that some of the previously unrecognized groups represent important components of soil microbial communities (7). Modification of the “universal” PCR primers of eukaryotes disclosed the importance of hidden groups by the previous approaches (47). Metatranscriptomic exploration also demonstrated the unexpected presence of typically marine and freshwater protists in soils (24). The HTS approaches enabled the surveys of soil protistan communities in remote, harsh, hitherto unsurveyed environments where culture-dependent approaches were not easily applicable (2, 15, 58). Publicly available metagenome data are less used for studying the diversity of soil microeukaryotes but can provide valuable information as they are free from the PCR biases and include the sequences of genes that are not targeted in amplicon-based approaches (34). The rhizosphere of plants is known to be a hot spot of soil protists (4, 5) and the phyllosphere may be another important habitat for protists in terrestrial ecosystems (52).

It becomes also evident that the geographical diversity of soil protists shows considerably different patterns from those of soil bacterial communities (7). The large-scale molecular data suggest that the protistan community patterns are highly consistent within habitat types and geographic regions and thus considered to reflect to their ecology in the environments (27).

In soil, protists mostly inhabit the water film and thus the soil water availability controls the protistan community in a global scale of geography with relation to the climatic conditions (7). Soil moistening can selectively enhance the growth of protists (20). Oxygen should be another important chemical factor to affect the protistan community structure in soil in particular with high water content such as submerged rice field soil (47). The soil protistan community structure also responds to a wide range of oxygen tension (61). Anoxia and hypoxia are not necessarily constraints for the growth of protists as demonstrated in aquatic environments (36). The predominance of active heterolobosean amoeba under anoxic conditions was demonstrated by RNA-based molecular analysis (47). Soil pH, an important environmental factor to shape the soil bacterial community, is known to affect the emerging abundance of putatively parasitic protists (17).

Soil management is an important anthropogenic force to affect the protistan community development. The soil nutrient status controls the community of testate amoebae which are among the most important and abundant protists in acidic forest ecosystems (38). Soil management practices in agriculture like fertilization and organic loading also alter the protistan community (47, 48). Fertilization can shape the structure of soil microbial food web (41, 48). Heat stress dramatically disturbs the ciliate community in the greenhouse soil (49).

With accumulation of sequence information in the public database, the community composition of specific groups of soil protists has been intensively studied using the specific primers for the target groups: e.g., Ciliophora (53, 59), Cercozoa (3, 41), Acanthamoeba (18), Kinetoplastea (41), and Apusomonads (62).

## A three-legged race with culture-dependent approaches

Protists remain difficult to discover and identify because of their small body sizes and patchy distributions, the low abundance of many species, and the difficulty in cultivation. Furthermore, the limited public interest in and knowledge of these organisms may hinder research progress in several protistological disciplines (29). It may sound like a paradox but is true that culture-independent molecular analyses of protists in environments highlight the importance of culture-dependent studies of protists. Challenge of cultivating new and poorly-studied organisms and integration of such cultivation techniques with molecular and high-end microscopical techniques will provide enormous insights into fundamental questions in protistology (29). Indeed, even for *Acanthamoeba*, a very common group of soil protists including relatively many isolates so far, the molecular approach has clarified that the diversity is still far from the full understanding (21). A recent HTS analysis of *Acanthamoeba* for 150 grassland soil samples has revealed that the 37% of 273 OTUs identified have similarities less than 96% with the known sequences (18).

## New ecological insights of soil phagotrophic protists

Phagotrophic protists play a crucial role as microbial grazers in soil ecosystems. Their selective grazing leads to the selection of the bacterial community structures in soil (44, 45, 54). Protistan grazing also alters the gene expression of prey bacteria, which affects the viability of trophozoites form of the grazer amoeba (60).

Recent studies have provided further insights of the prey-predator interactions. The trophic interaction would be highly associated with the coexisting bacterial diversity and function (10). The effect of protistan grazing on the virulence of opportunistic pathogenic bacteria is related with the coexisting bacteriophages (19). In addition, amoeba need some vitamins produced by coexisting heterotrophic bacteria to graze on cyanobacteria (43)

Furthermore, recent findings have demonstrated that the ecological functions of phagotrophic protists are more diverse than recognized before (25, 63). A newly isolated novel species of soil testate amoeba does not graze on bacteria but on algae and fungi (16). Testate amoeba even have a strategy of pack hunting on bacterivorous nematodes (23). Flagellates also attack nematodes (9). Protists can be parasites of soil Metazoa (22) and also hosts of novel bacteria and archaea (30–32, 35, 66). Stable isotope probing of microorganisms in detritusphere demonstrated the significant role of trophic interaction and succession of microorganisms where protists are involved as bacterivores, fungivores, and even saprotrophs. (37, 46).

These findings have renewed the conventional concept of the soil microbial food web and indicated that the soil microbial food web is much more complicate than previously recognized. Further investigation is needed to understand the functional roles of protists in soil ecosystems.

## Sitting on a gold mine

Molecular approaches have opened the gate of a new era in soil protistology. The results should be, however, interpreted with cautions of the limitation and drawbacks embraced in the techniques. Technical issues in molecular analyses of soil protists remain to be solved (56, 57). There are partly incompatible databases present (17). The HTS does not always give us a true picture of protistan community (22). The international initiative to build a universal taxonomic framework for eukaryotes has launched to bridge the protist-omics age to the fragile, centuries-old body of classical knowledge (8). The similar activity for ciliates has also just started (64).

New molecular techniques are gradually available in protistology. Single cell-based genomics (40, 59) and transcriptomics (42) were applied to different types of single cellular eukaryotes including protists. Genome editing by CRISR/Cas9 should be frequently used for the study of the protistan physiology and biochemistry (39).

The International Society of Soil Protistologists has recently proposed common questions to be answered after the extensive survey (26), which clearly states how little we know about soil protists—that means how many scientific treasures are buried under our feet. Folks, it is time to go hunting with the new map, compass, and shovel!
